# Characterization of an HNA aptamer suggests a non-canonical G-quadruplex motif

**DOI:** 10.1093/nar/gkad592

**Published:** 2023-07-13

**Authors:** Peter Schofield, Alexander I Taylor, Jérôme Rihon, Cristian D Peña Martinez, Sacha Zinn, Charles-Alexandre Mattelaer, Jennifer Jackson, Gurpreet Dhaliwal, Guy Schepers, Piet Herdewijn, Eveline Lescrinier, Daniel Christ, Philipp Holliger

**Affiliations:** Garvan Institute of Medical Research, Darlinghurst, Sydney, NSW 2010, Australia; St Vincent's Clinical School, Faculty of Medicine, University of New South Wales, Kensington, Sydney, NSW 2010, Australia; MRC Laboratory of Molecular Biology, Cambridge CB2 2QH, UK; Cambridge Institute of Therapeutic Immunology & Infectious Disease (CITIID), University of Cambridge, Cambridge CB2 0AW, UK; Rega Institute, Laboratory of Medicinal Chemistry, Katholieke Universiteit Leuven, Herestraat 49, B 3000, Leuven, Belgium; Garvan Institute of Medical Research, Darlinghurst, Sydney, NSW 2010, Australia; St Vincent's Clinical School, Faculty of Medicine, University of New South Wales, Kensington, Sydney, NSW 2010, Australia; Garvan Institute of Medical Research, Darlinghurst, Sydney, NSW 2010, Australia; St Vincent's Clinical School, Faculty of Medicine, University of New South Wales, Kensington, Sydney, NSW 2010, Australia; Rega Institute, Laboratory of Medicinal Chemistry, Katholieke Universiteit Leuven, Herestraat 49, B 3000, Leuven, Belgium; Garvan Institute of Medical Research, Darlinghurst, Sydney, NSW 2010, Australia; Cambridge Institute of Therapeutic Immunology & Infectious Disease (CITIID), University of Cambridge, Cambridge CB2 0AW, UK; Rega Institute, Laboratory of Medicinal Chemistry, Katholieke Universiteit Leuven, Herestraat 49, B 3000, Leuven, Belgium; Rega Institute, Laboratory of Medicinal Chemistry, Katholieke Universiteit Leuven, Herestraat 49, B 3000, Leuven, Belgium; Rega Institute, Laboratory of Medicinal Chemistry, Katholieke Universiteit Leuven, Herestraat 49, B 3000, Leuven, Belgium; Garvan Institute of Medical Research, Darlinghurst, Sydney, NSW 2010, Australia; St Vincent's Clinical School, Faculty of Medicine, University of New South Wales, Kensington, Sydney, NSW 2010, Australia; MRC Laboratory of Molecular Biology, Cambridge CB2 2QH, UK

## Abstract

Nucleic acids not only form the basis of heredity, but are increasingly a source of novel nano-structures, -devices and drugs. This has spurred the development of chemically modified alternatives (xeno nucleic acids (XNAs)) comprising chemical configurations not found in nature to extend their chemical and functional scope. XNAs can be evolved into ligands (XNA aptamers) that bind their targets with high affinity and specificity. However, detailed investigations into structural and functional aspects of XNA aptamers have been limited. Here we describe a detailed structure-function analysis of LYS-S8-19, a 1′,5′-anhydrohexitol nucleic acid (HNA) aptamer to hen egg-white lysozyme (HEL). Mapping of the aptamer interaction interface with its cognate HEL target antigen revealed interaction epitopes, affinities, kinetics and hot-spots of binding energy similar to protein ligands such as anti-HEL-nanobodies. Truncation analysis and molecular dynamics (MD) simulations suggest that the HNA aptamer core motif folds into a novel and not previously observed HNA tertiary structure, comprising non-canonical hT-hA-hT/hT-hT-hT triplet and hG4-quadruplex structures, consistent with its recognition by two different G4-specific antibodies.

## Graphical Abstract

**Graphical Abstract:**
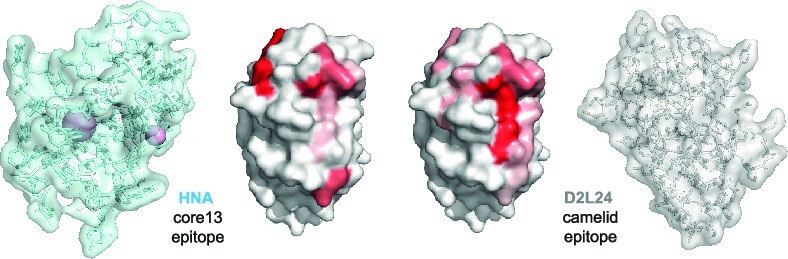

## INTRODUCTION

Nucleic acids are an unique form of sequence-defined polymer encoding both genetic information (i.e. the genotype) as well as functional information (i.e. the phenotype), and can therefore be evolved directly at the molecular level. However, the functional scope of nucleic acid-based polymers is limited by systemic shortcomings inherent in nucleic acid chemistry. These include comparative chemical uniformity and consequently a narrow range of physicochemical properties (dominated by the polyanionic phosphodiester backbone) and limited biostability due to the ubiquitous presence of powerful nucleases. This has spurred the development of chemical alternatives to the natural nucleic acids, also called xeno-nucleic acids (XNAs), which provide a wider range of chemical and biophysical properties and - in some cases - higher biostability (1). Together with the engineering of bespoke polymerases for template-instructed XNA synthesis and reverse transcriptases (RTs) for their reverse transcription back into DNA, this has enabled the selection and *in vitro* evolution of XNA ligands (aptamers) and catalysts (XNAzymes) directly from diverse repertoires of a variety of XNAs bearing modified or alternative backbones, sugars or congeners as well as the elaboration of XNA nanostructures (2–6).

**Figure 1.**
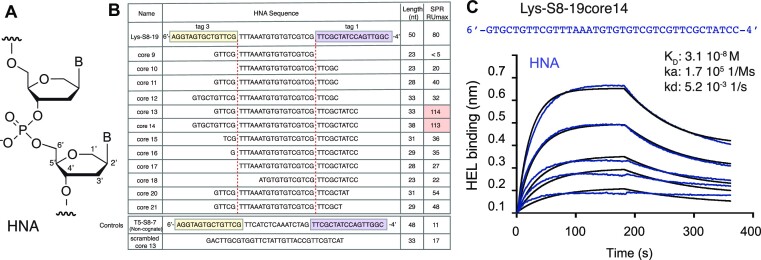
Aptamer truncation analysis. (**A**) Structure of 1′,5′ anhydrohexitol nucleic acids (HNA). (**B**) Truncation of analysis of 50 nt Lys-S8-19 with aptamer length and HEL binding (SPR RU max) given showing that 38 nt Lys-S8-19-core14 and 33 nt Lys-S8-19-core13 truncations not only retain but enhance HEL binding. (**C**) Affinity analysis of chemically synthesized Lys-S8-19-core14 binding to hen egg-white lysozyme (HEL) by biolayer interferometry (BLI). Binding of soluble core14 was measured on streptavidin biosensors loaded with biotinylated HEL. Global fitting of kinetics revealed a binding affinity of 31 nM.

Among the first XNAs to be evolved, were aptamers composed entirely of the unnatural nucleic acid 1′,5′-anhydrohexitol nucleic acid (HNA), in which the canonical five-membered ring of ribofuranose is replaced by a six-membered anydrohexitol ring (Figure 1A). The selected HNA aptamers were found to bind specifically either to the HIV-TAR RNA motif or hen egg white lysozyme (HEL) with affinities in the nanomolar range (3). Concurrently, anti-thrombin aptamers composed of a 3-letter α-l-threofuranosyl nucleic acids (TNA) alphabet were described(7). More recently, and building on improved XNA polymerases and selection methodologies, a wide range of XNA aptamers have been discovered, notably 2′-fluoroarabino nucleic acid (FANA) and TNA aptamers against HIV reverse transcriptase (HIV RT) (8–10) and HIV integrase (11). Furthermore, HNA (12), 2′F-RNA (13) and 2′OMe (2′-*O*-methyl)-RNA (14,15) and mixed 2′OMe-/MOE (2′-*O*-(2-methoxyethyl))-RNA aptamers to vascular endothelial growth factor (VEGF) (16) and anti-streptavidin aptamers in an uncharged P-methyl-/P-ethyl-phosphonate backbone (phNA) (17) have been discovered. In addition to proteins, XNAs can also form receptors for small molecule ligands, as shown by the discovery of a TNA aptamer specific for ochratoxin A (18).

However, while such XNA aptamers have been characterized at the level of sequence, function and secondary structure little is known about characteristics of their binding sites (paratopes), and about their folding and structural traits, other than that they are likely to diverge substantially from standard nucleic acids based on the structural diversity uncovered in structures of short XNA duplexes (19). Furthermore, the accuracies of XNA secondary structure predictions are unclear, as they are generally based on DNA or RNA parameters, which may diverge to a large extent depending on the type of XNA. Here, we have examined one of the first XNA aptamers described (the anti-hen egg-white lysozyme HNA aptamer LYS-S8-19) (3) in detail, by carrying out structure-function studies, molecular dynamics simulation and comparative analyses with well-characterized anti-HEL immunoglobulin antibodies.

## MATERIALS AND METHODS

### HNA oligonucleotide synthesis

HNA oligonucleotide synthesis was performed essentially as described (20). Oligonucleotide assembly was performed on an Expedite^®^ DNA synthesizer (Applied Biosystems) by using the phosphor-amidite approach. The HNA assembly protocols were adjusted for MMTr detritylation, coupling, capping and oxidation. The coupling time was changed to 8 min. The HNA oligomers were deprotected and cleaved from the solid support by treatment with AMA solution (half part composed of ammonia 33% and half of methylamine 40%) for 2 h at 35°C. After gel filtration on NAP-25^®^ column (Sephadex G25-DNA grade; Pharmacia) with acetonitrile (20%), the crude mixture was analyzed on a Mono Q ™ 10/100 column (Pharmacia) ion exchange column (pH 12), after which purification was achieved on the same instrument with the following gradient system (A = 10 mM NaOH, pH 12.0, 0.1 M NaCl with 15% acetonirile; B = 10 mM NaOH, pH 12.0, 0.9 M NaCl with 15% acetonitrile) and lyophilized. The liquid chromatography system consisted of a Primaide-Hitachi 1110 pump system, a Mono Q™ 10/100 column (Pharmacia). The product-containing fraction was desalted on a NAP-25^®^ column and lyophilized and mass analysed to test purity (Supplementary Figure S1).

### Preparation of HNA aptamer variants

Triphosphates of HNA (hNTPs) were synthesized according to established protocols (21). DNA oligonucleotides were synthesized by IDT (Integrated DNA technologies BVBA, Leuven, Belgium)(Supplementary Table S1). Polymerases were prepared and HNA was synthesized as described previously(3,4) using HNA polymerase 6G12 I521L. Briefly, 100 μl reactions were prepared using 1 μM primer (‘Cy3Fd’), 2 μM template (e.g. ‘LYS-S8-19temp’), 125 μM of each hNTP in 1x ThermoPol buffer in a two-step extension (60 min 50°C, 120 min 65°C). For truncation experiments, DNA templates and primers were digested following HNA synthesis using DNase I: reactions were diluted into 1× DNase buffer (New England Biolabs Inc. (NEB), Massachusetts, USA), incubated with 10 U TurboDNase (Ambion Inc., Texas, USA) for 2 h at 37°C.

For all other experiments, HNA was synthesized using 3′ biotinylated DNA templates (e.g. ‘LYS-S8-19-core14[wt]temp’) and primer ‘Cy3Fd_1R’, bearing a single 3′ RNA residue; templates were removed using MyOne Dynabeads Streptavidin C1 magnetic beads (Thermofisher, USA), and primers removed by alkaline hydrolysis (0.8N NaOH, 65°C, 1 h). All HNA aptamers were purified by urea-PAGE and extracted from gel slices by maceration, freeze-thawing, passive diffusion (overnight at room temperature) and filtration though a 0.2 μm Spin-X column (Corning Life Sciences, Massachusetts, USA), then ethanol precipitated. Before use, HNA aptamers were resuspended in nuclease-free water (Qiagen, UK) and concentrations were determined using a nanodrop instrument (Thermo Fisher Scientific, Massachusetts, USA).

### Measurement of binding of truncated aptamer variants to HEL

PAGE-purified variants of LYS-S8-19 were annealed at 1 μM in nuclease-free water by rapid heating and cooling (2 min 94°C, 10 min 17°C) and analysed in buffer R: 20 mM HEPES (pH 7.4), 20 mM NaOAc, 140 mM KOAc, 3 mM Mg(OAc)_2_, 0.1% (v/v) Tween20. Surface plasmon resonance (SPR) measurements were made using a BIAcore 2000 instrument (GE Healthcare UK Ltd, UK) at a flow rate of 20 μl min^−1^ in buffer R at 20°C. CM4 sensor chip (GE Life Sciences UK Ltd, UK) surfaces were coated with Neutravidin (Pierce 31000, Thermo Fisher Scientific, Massachusetts, USA) surfaces (∼10 000 RU per flow cell) using an amine coupling kit (GE Life Sciences UK Ltd, UK) and flowing in 5 mM NaOAc (sodium acetate), pH 5.5. Chips were equilibrated in buffer R and left to flow overnight until signal drift had settled. ∼2000 RU biotinylated hen egg lysozyme (Sigma Aldrich LO289; Sigma-Aldrich Company Ltd, UK) was captured (except for the reference cell) before blocking with excess free biotin. 50 μl 200 nM LYS-S8-19 variants were injected for 150 s, in buffer R. After every injection, the sensor surface was regenerated using two 5 μl injections of 50 mM NaOH, 1 M NaCl.

### Anti-lysozyme antibodies, anti-motif antibodies and alanine scan mutants of HEL

Anti-Lysozyme V_HH_ domains D2L24, D2L19 and D3L11 were previously described (22). Selection of anti-HEL human V_H_ (H04 and D05) and V_L_ (VL5 and VL12) domains was described previously (23,24). Expression of all V_HH_, V_H_, V_L_ and scFv antibody fragments was performed using pET12a periplasmic expression vector (Novagen) in BL21-Gold (DE3) (Agilent) and subsequent purification with protein A or L sepharose (GE) was performed as previously described (23,24). Anti-G4 motif antibody BG4 (25) was purchased in an IgG format (Absolute Antibody Ab00174-1.1).

Selection of the anti-G4 Gmab scFv was carried out by phage display as described previously (26) and expressed in *Escherichia coli* bacteria (27). Binding specificity of Gmab to G4 quadruplex by BLI as demonstrated in Supplementary figure 5. Anti-HEL mouse monoclonal antibodies HyHEL10 and HyHEL5 were expressed in Expi293 expression system (Thermo Fisher Scientific) and purified by His tag as previously described (28). Alanine substitution mutant variants of Hen Egg Lysozyme were generated by de novo gene synthesis (Genscript) then expressed using the Expi-293 expression system and purified by affinity chromatography as previously described (28). Wild-type HEL was sourced commercially (Sigma).

### Affinity measurements of aptamer binding to HEL and various antibodies

Binding affinity of soluble Core14/Core13 aptamers with HEL and various antibodies was determined by BioLayer Interferometry (BLItz, Fortebio) using the advanced kinetics program on the BLItz Pro 1.2 software. Lysozyme and purified antibodies were biotinylated using EZ-Link NHS-PEG4-biotinylation reagent as per the manufacturer's instructions (Thermo Fisher Scientific). Streptavidin Biosensors (Fortebio), biotinylated proteins and Core14/Core13 aptamer were all reconstituted in R buffer (20 mM NaOAc, 140 mM KOAc, 3 mM MgOAc_2_, 20 mM HEPES pH 7.5, 0.1% Tween). Biotinylated proteins were then loaded onto streptavidin biosensors for 180 s followed by a 30 s baseline in R buffer. The protein-loaded sensors were then incubated for 180 s with shaking in a range of concentrations of Core14/Core13 aptamer to measure binding association. Dissociation was then measured by placing the biosensors in a fresh tube of R-buffer for a further 180 s. Binding kinetics were fit globally for binding curves observed at four or more aptamer concentrations of aptamer using the BlitzPro 1.2 software. Binding kinetics for anti-motif monoclonals against G4 quadruplex or i-motifs were measured using a similar protocol. For measurement of anti-G4 binding in the presence or absence of different ions R-buffers were as follows: Buffer R_Na+_ (w/o K+): 20 mM HEPES (pH 7.4), 160 mM NaOAc, 3 mM Mg(OAc)_2_, 0.1% (v/v) Tween20, Buffer R-Mg (w/o Mg^2+^): 20 mM HEPES (pH 7.4), 20 mM NaOAc, 140 mM KOAc, 5 mM EDTA, 0.1% (v/v) Tween20, Buffer R_Li+_ (w/o K^+^): 20 mM HEPES (pH 7.4), 160 mM Li acetate, 3 mM Mg(OAc)_2_, 0.1% (v/v) Tween20. The relevant BLI data is always given (KD, Ka, Kd) and KD is a composite of multiple (at least *n* = 5, for figures in this manuscript and *n* = 4 for the supplementary tables).

### Binding competition assays

The Core14 binding epitope for HEL was also determined by BLI using binding competition assays with anti-HEL antibody fragments. As above, all streptavidin biosensors, aptamer, and proteins were equilibrated in R buffer (20 mM NaOAc, 140 mM KOAc, 3 mM MgOAc_2_, 20 mM HEPES pH 7.5, 0.1% Tween). Assays were run on the BLItz system using the advanced kinetics program on the BLItzPro 1.2 software. Streptavidin biosensors were first loaded with biotinylated HEL for 180 sec. A first association step was then carried out where a high concentration (e.g. 4 μM) of unbiotinylated anti-HEL antibody fragment was allowed to bind the sensor-loaded HEL to saturation for 120 sec. The sensor was then immediately placed in a second 120 sec association containing the same concentration of anti-HEL antibody fragment with the addition of 2 μM Core 14 aptamer. Amplitude of the Core 14 aptamer binding curve in the presence of antibody was compared to the magnitude of aptamer binding curve in the absence of competitor antibody. HEL epitope binding competition was determined for each antibody as a percentage loss of aptamer binding curve magnitude.

### Epitope mapping by alanine scan

Finer epitope mapping of the Core14 interaction with HEL were performed on the BLItz using an advanced kinetics program on the BLItz Pro 1.2 software. Mutant HEL variants baring single alanine substitutions for amino acid residues within the characterised V_HH_ D2L24 binding epitope were biotinylated as described above and loaded to an amplitude of 4 nm onto streptavidin sensors equilibrated in R buffer. Sensors were then incubated with 100nM Core14 aptamer for 180 s. Magnitude of the binding curves was compared with the binding curve of 100 nM Core14 vs biotinylated WT HEL. Crucial residues for aptamer binding were determined as those causing the greatest percentage loss in binding curve amplitude when substituted for alanine.

### MD simulations

All simulations were performed using AMBER18 (29). The exploratory replica exchange molecular dynamics (REMD) simulation was performed for 1 μs with an exchange attempt frequency of 0.1 ps^−1^ (timestep 0.002 fs). A total of 20 replicas were spaced between a simulation temperature of 300 and 506 K (30). Langevin dynamics with a collision frequency of 1 ps^−1^ was employed while applying SHAKE(31) to restrain bonds involving hydrogen atoms. Implicit solvation^[4]^ with a theoretical salt concentration of 0.2 M was included.

The proposed model itself was generated using an iterative simulated annealing (SA) approach where additional restraints were included in each step. Each SA step consisted of an equilibration step at 300 K (5 ps), short heating to 500 K (5 ps), a longer cool down period to 300 K (170 ps) and finally another equilibration at 300 K (20 ps). The timestep was set to 0.002 ps per MD step. Langevin dynamics with a collision frequency of 1 ps^−1^ was employed while applying SHAKE (31) to restrain bonds involving hydrogen atoms.

The initial model was generated after eight rounds of SA (Supplementary Table S2) where interatomic distance restraints together with dihedral restraints were arbitrarily chosen to prevent the formation of steric clashes due to restraints. The first six rounds added restraints cumulatively. In the eight rounds of SA, planarity restraints were additionally applied on residues 1–13-20–23. A force constant of 5 kcal/mol/Å² was employed for the restraints on interatomic distances and 20 kcal/mol/rad² for the dihedral restraints. After those eight rounds, we noticed a strained conformation of the TAT triplet, therefor a next round of SA was performed using XPLOR where the orientation of the base pairing between the adenosine and thymidine residues was swapped.

The initial model was relaxed within the conformational space governed by the set restraints in a REMD experiment for 400 ns. The restrained REMD was setup identically as previously described, only adjusting the number of replicas (16) and reducing the exchange attempts to reduce the simulation time. After cluster analysis, the structure with the lowest energy in the implicit solvent was chosen for the prolonged MD simulation in explicit solvent. A K^+^ ion was placed at the center of the Gq structure in the initial model and a Mg^2+^ ion was placed between phosphate moieties of hG20 and hT12, four Na^+^ and 26 K^+^ ions were added to represent experimental conditions and neutralize the molecule. 5646 TIP3P water molecules were added in a truncated octahedron that extends 12 Å from the molecule. Prior to the 1 μs unrestrained MD, a 50ns restrained MD was performed with restraints on the residues of the Gq, the TAT triplet and the two stabilizing ions. For all simulations, a cutoff value of 10 Å was used for long range and electrostatic interactions.

The solvated molecule was minimized using 10 000 steps of steepest descent followed by 40 000 steps of conjugate gradient minimization. Langevin dynamics with a collision frequency of 1 ps^−1^ was employed for all subsequent MD simulations while applying SHAKE^14^ to restrain bonds involving hydrogen atoms. After the minimization, the system was heated to 300 K in 50 ps (timestep 0.002 ps) while keeping the volume constant. The density was equilibrated afterwards in 1 ns (timestep 0.002 ps) while keeping the pressure constant. The restraints were subsequently reduced over the course of 50 ns (timestep 0.002 ps). Finally, a 1 μs production MD (timestep 0.002 ps) without any restraint was performed to check the stability of the proposed model. A mutant was run under the same condition in explicit solvent, the G27A variant, after the final HNA aptamer model was conceived, to discuss its biological implication (Supplementary Figure S4).

## RESULTS

Anti-HEL aptamers were previously isolated from an all-HNA library of ≈ 10^15^ unique sequences, by selection for binding to biotinylated HEL immobilized on streptavidin-coated magnetic beads(3). Sequencing of hits revealed a common but unusual GT-rich core motif with variable 6′ and 4′ extensions (Supplementary Figure S2). Among these LYS-S8-19 bound HEL with high nanomolar affinity *in vitro* or when the antigen was displayed on the surface of cells, and was found to be specific for HEL, with little or no binding to unrelated protein targets(3).

### Identification of a truncated HNA aptamer motif

The full-length LYS-S8-19 sequence (50 nt) comprises a truncated 18 nt ‘core’ sequence derived from the degenerate (N_40_) region of the HNA library used during aptamer selection(3). This core is flanked by two sequences used as subsequent PCR priming sites during the selection; ‘tag1’(17 nt) and ‘tag3’(15 nt). In order to better define the functional aptamer motif of LYS-S8-19, we prepared a series of variants with systematically truncated 6′ or 4′ termini (Figure 1b), synthesized them enzymatically using polymerase 6G12 I521L(4) and measured their binding to immobilized HEL using surface plasmon resonance (SPR). This showed that both tag sequences contribute to aptamer folding and/or binding as their complete deletion led to loss of HEL binding. Nevertheless, the main HEL binding paratope appears to be located in the core as the larger part of tag1 & tag3 sequences could be truncated with broad retention of HEL binding (Figure 1b) requiring just over five nucleotides from each tag sequence to retain above-background binding (as defined by a non-cognate HNA aptamer ‘T5-S8-7' (specific for TAR RNA)(3), or a scrambled LYS-S8-19 variant). The shortest variants measured that retained full HEL binding (in fact, with higher affinity than that of the full-length sequence) was found to be LYS-S8-19-core13 (33 nt) and LYS-S8-19-core14 (38 nt) (Figure 1B). The shortest variant that still retained clear HEL binding above background was LYS-S8-19-core21 (29 nt), which comprises the core sequence flanked by just 6 residues from tag1 and 5 from tag3. Non-specific binding to the blank reference surface (neutravidin without biotinylated HEL) used in the SPR assay could be observed with LYS-S8-19-core9 and -core13 (and to a lesser extent, -core15 and -core16) indicating a folding defect (Supplementary Figure S2). We decided to focus on LYS-S8-19-core14 and LYS-S8-19-core13 as the truncated HNA aptamers in subsequent experiments.

### Chemical synthesis and characterization of truncated HNA aptamers

Truncated HNA aptamer LYS-S8-19 (LYS-S8-19-core14 (henceforth called core14)) was chemically synthesized (20) (see Materials and Methods) using HNA phosphoramidates and its affinity for HEL was determined by biolayer interferometry (BLI) (Figure 1C). The chemically synthesized HNA aptamer bound to HEL with nanomolar affinity (*k*_a_ = 1.7 × 10^5^ s^−1^M^−1^, *k*_d_ = 5.2 × 10^−3^ s^−1^, *K*_D_ = 31 nM), approximately three- to four-fold tighter than the enzymatically synthesized full-length HNA aptamer determined previously by BLI and SPR(3).

### Mapping the core14/HEL binding interface through competition

**Figure 2.**
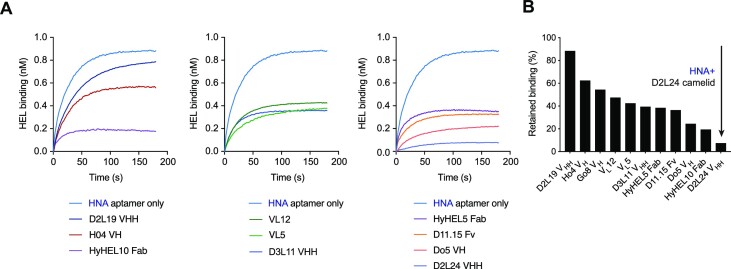
LYS-S8-19-core14 epitope mapping. Mapping the core14 binding site on HEL by binding competition on BLI: (**A**) core14 binding curves to streptavidin biosensors loaded with HEL-biotin and pre-incubated with various HEL binding antibody fragments. (**B**) Histogram representation of retained binding by core14 to HEL-loaded biosensors pre-incubated with competitors – calculated as magnitude of Rmax compared to the Rmax of core14 binding curve in the absence of competitor. Lower values indicate greater competition between antibody and aptamer for HEL binding epitope.

As HEL is a protein with significant positive charge potential (p*I*) and positively charged surface regions, HEL binding by a ligand with a polyanionic phosphodiester backbone is likely to contain a significant electrostatic component. Indeed, previously described anti-HEL aptamers showed much reduced binding at higher ionic strength(32). We sought to minimize such non-specific binding in the case of the anti-HEL HNA aptamers by conducting both selections and characterization at near-physiological ionic strength. To investigate the nature and specificity of the core14 HNA aptamer HEL interaction, we performed competition experiments using a range of previously reported antibody reagents specific for HEL, for which crystal structures had been reported: the set included a range of antibody formats, including Fab (HyHEL10, HyHEL5)(33,34), VHH camelid domains (D2L19, D3L11, D2L24) (22) and human VH and VL single domains (H04, VL12, VL5, D05) (23,35), as well as a Fv fragment (D11.15) (36). These experiments indicated specific interaction of core14 with HEL, with the aptamer competing for HEL binding with only a specific subset of antibody reagents as would be expected for a specific interaction with a spatially defined HEL epitope. Specifically, core14 competed strongly with Fab HyHEL10 and in particular with the camelid domain VHH D2L24 for binding (Figure 2).

In contrast, the aptamer did not compete with other antibody reagents such as the H04 human single VH domain which predominantly interacts with lysozyme through its active site cleft (23). These interaction analyses thereby broadly define the epitope of HNA aptamer binding on the HEL protein surface.

### Alanine scanning analysis of the convergent HEL D2L24 and LYS-S8-19-core14 interface

**Figure 3.**
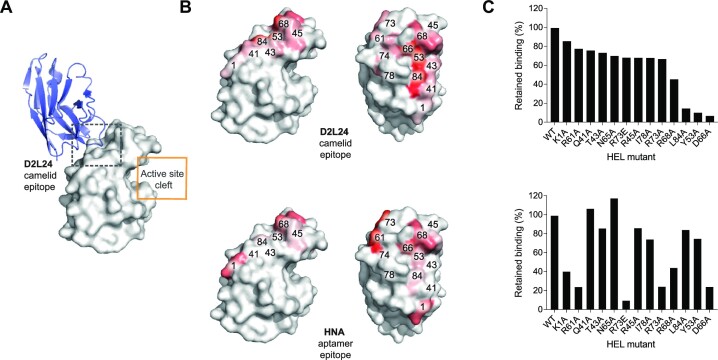
Fine mapping of binding epitopes. Alanine scan of core14 and D2L24 binding epitopes on HEL: (**A**) crystal structure of the D2L24:HEL complex. D2L24 binding epitope is shown on opposite side of HEL in comparison to the HEL active site cleft. (**B**) Heat map representation of HEL alanine mutations affecting D2L24 or core14 binding. Variants of HEL with alanine substitutions at the residues specified were biotinylated and loaded onto streptavidin BLI biosensors. D2L24 and core14 binding curve magnitudes against HEL variants (Rmax) were compared to those observed for WT HEL. Increased intensity of red coloration indicates higher effect of alanine residue on binding. (**C**) Histogram representation of retained binding to HEL mutants (Rmax of HEL alanine variant over Rmax of WT HEL).

Next, to gain a more detailed understanding of the interaction epitope and interface, we fine mapped the interface based on the co-crystal structure of D2L24 in complex with lysozyme (Figure 3A) (22). Single alanine and charge reversion mutations were introduced into HEL at positions in the vicinity of the D2L24 contact interface. Specifically, positions 1, 41, 43, 45, 53, 65, 66, 68, 73, 74, 78 and 84 were targeted for substitution with alanine and the resulting mutant HEL variants expressed by secretion in HEK293 cells (Supplementary Methods). Fine mapping of the D2L24 HEL epitope revealed a predominantly flat epitope, adjacent but distinct from the lysozyme active site cleft (Figure 3B).

In a next step, the same set of mutants was tested for binding to core14. This revealed an epitope broadly overlapping with the D2L24 epitope with both interactions centred on hotspot residues at, and adjacent to, HEL position 66 (Figure 3B, C). Thus, core14 and the VHH camelid domain D2L24 share a common binding epitope and interaction hotspot on HEL in agreement with binding competition experiments (Figure 2). Despite overlapping epitopes, mutation analysis also indicates some differences in interaction modes. While HNA is more hydrophobic than DNA (37), it is noticeable that core14 HNA aptamer binding is more dependent on ionic interactions (with positively charged amino acids e.g. K1, R61, R73) in the HEL epitope than the antibody VHH domain.

### Probing of aptamer structure with monoclonal antibodies

**Figure 4.**
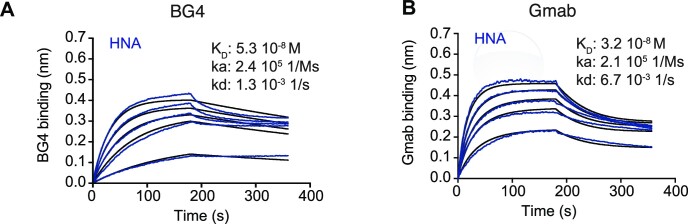
HNA quadruplex recognition by anti-G4 antibodies. (**A**, **B**) BLI affinity analysis of soluble core14 binding to biotinylated anti-G quadruplex antibodies BG4 (A) and Gmab (B) (see also Supplementary Figure S5) for specific DNA G4 recognition versus i-motif binding

To further probe the HNA aptamer structure, we analyzed binding of core14 to conformation-specific antibodies specifically the previously reported (and well-characterized) anti-G4 DNA quadruplex monoclonal antibody BG4 (25). As a negative control, we used 4D5 Fab, a common model antibody derived from the human antibody therapeutic Herceptin (38). These experiments revealed that core14 indeed binds to BG4 with nanomolar affinity (Figure 4A), while no detectable binding was observed for 4D5.

To further validate the observed cross reactivity observed with BG4 and rule out the possibility that G4 recognition in the HNA aptamer is an artefact of BG4 cross-reactivity, we used the Garvan-2 human single-chain variable fragment (scFv) library to select novel anti-DNA G4 quadruplex specific antibody fragments (23,39,40). Phage selections were performed against G4 quadruplex DNA and binders identified using soluble fragment enzyme-linked immunosorbent assay (ELISA), followed by bio-layer interferometry (BLI) (26). This approach resulted in the identification of a candidate (Gmab), which was expressed in *E. coli* in an scFv antibody fragment format. We first evaluated the specificity of the selected antibody fragment for DNA G4 quadruplex DNA; this analysis revealed specific and high-affinity binding of Gmab to G4 quadruplex DNA (KIT2) but not to i-motif control DNA (iM stable) (Supplementary Figure S5). Similar G4 quadruplex DNA binding specificity was observed for BG4 ((25) and Supplementary Figure S5). We observed stable complexes with both anti-G4 antibodies and core14 HNA aptamer, characterized by a slow dissociation rate and *K*_D_ in the nanomolar range for both BG4 (*k*_a_ = 7.9 × 10^4^ s^−1^M^−1^, *k*_d_ = 1.5 × 10^−4^ s^−1^, *K*_D_ = 1.9 nM) and Gmab (*k*_a_ = 7.5 × 10^4^ s^−1^M^−1^, *k*_d_ = 2.7 × 10^−4^ s^−1^, *K*_D_ = 3.6 nM) binding to G4 quadruplex DNA (Supplementary Figure S5) as well as for binding to core14 (BG4: *k*_a_ = 2.4 × 10^5^ s^−1^M^−1^, *k*_d_ = 1.3 × 10^−3^ s^−1^, *K*_D_ = 5.3 nM Gmab: *k*_a_ = 2.1 × 10^5^ s^−1^M^−1^, *k*_d_ = 6.7 × 10^−3^ s^−1^, *K*_D_ = 32.3 nM) (Figure 4A, B). In summary, in addition to binding to G4 quadruplex DNA, BLI analysis demonstrated binding by both BG4 and Gmab antibodies to core14 (Figure 4), strongly suggesting (i) that the HNA aptamer adopts a G4 quadruplex or G4 quadruplex-like structure and (ii) that the epitope recognized by both antibodies is largely independent of sugar (or congener) configuration in the nucleic acid backbone, but rather comprises a conformational epitope of nucleic acid strand folding (and presumably the positioning of guanine bases and phosphodiester groups). Next, we investigated HEL- and anti-G4 antibody (Gmab) binding to core14 and a selection of core13 mutants. These experiments suggested that residues hG1, hG13, hG20 and G23 (but not hG27) were essential for anti-G4 antibody (Gmab) binding (Supplementary Table S3), presumably defining the putative G4 structure. More detailed investigation of HEL-binding suggested that residues hT3, hA11 and hT29 were also needed for core13 HEL binding activity either as part of the HEL binding paratope or by involvement in structures needed for aptamer structure folding.

### Molecular dynamics simulations and formation of a G4 quadruplex structure

Unlike DNA and RNA, which have been shown to adopt extensive structural and topological diversity (including among others B-form DNA, zDNA, G4 quadruplex and i-motif structures) (41,42), little is known about the conformational, energetic and structural landscape of XNA oligomers beyond the structures of short oligomers (19). In particular, there is a lack of information on the structures adopted by functional XNA sequences such as XNA aptamers(1,3) or XNAzymes(4,43). All HEL binding HNA aptamers isolated in the original experiment displayed a striking sequence core motif rich in hG, hT residues (3) (Supplementary Figure S2) suggesting that hG, hT residues may be involved in the formation of a specific 3D-fold or structure critical for HEL binding. Probing of the core14 structure with anti-G4 antibodiesBG4 and Gmab (Figure 4) suggested that this structure likely accommodates an hG4 quadruplex, which we attempted to confirm by structure determination. However, our efforts at obtaining crystals of either the HNA aptamer alone or in complex with HEL were not successful and NMR structure determination on a natural abundance sample was unfeasible due to high signal overlap. We therefore turned to *in silico* modelling, specifically a combined approach of replica exchange molecular dynamics (REMD) and simulated annealing (SA) in implicit solvent using a modified version of the OL3-forcefield for RNA that was employed to correct for the extra methylene group in the HNA hexitol moiety and applied it to the smaller, computationally more tractable core13, which retains full HEL-binding activity (Figure 1b). Unrestrained REMD yielded 8 apparent clusters. In the most abundant structural cluster obtained after unrestrained REMD, a stem-loop with hG1-hG13 as the terminal base pair in the stem occurs (structural cluster c0). This secondary structural element is also found in three other clusters that show a highly similar secondary fold (c2, c4, c5). Based on the sequence, five residues (hG15, hG17, hG20, hG23, hG27) could be the remaining partners in the G-quartet identified in the biological assay. Since a single residue loop is unfeasible, hG15 was not considered as a possible candidate. In none of the obtained structures, hG17 occurs in close distance to the hG1-hG13 base pair. In the most populated cluster c0, hG20 is base pairing with the nucleobase of hG13. This leaves 2 residues for the remaining position in the G-quartet. Initially our focus was on quartet formed by hG1, hG13, hG20 and hG27 since in some structures of cluster c0 hG27 and hG20 came in the vicinity of the hG1-hG13 base pair and hG23 was excluded as additional stability of loops with minimum three residues was assumed for HNA as for described natural nucleic acids(44). However binding results of aptamer variants against anti-G4 DNA quadruplex monoclonal antibody Gmab revealed that hG23 was the fourth partner in a G-quartet instead of hG27 (Supplementary Table S3). Based on these data, a starting model was generated by an iterative simulated annealing approach over various steps with hG1, hG13, hG20 and hG23 in a G-quartet to replace the initial linear oligonucleotide. Each of these steps included additional restraints (Supplementary Table S1). The model obtained by simulated annealing was first subjected to a 50 ns restrained MD experiment in explicit solvent to allow structural relaxation. Next, the structure was submitted for an unrestrained MD simulation in explicit solvent for 1 μs in the presence of ions to check the stability.

**Figure 5.**
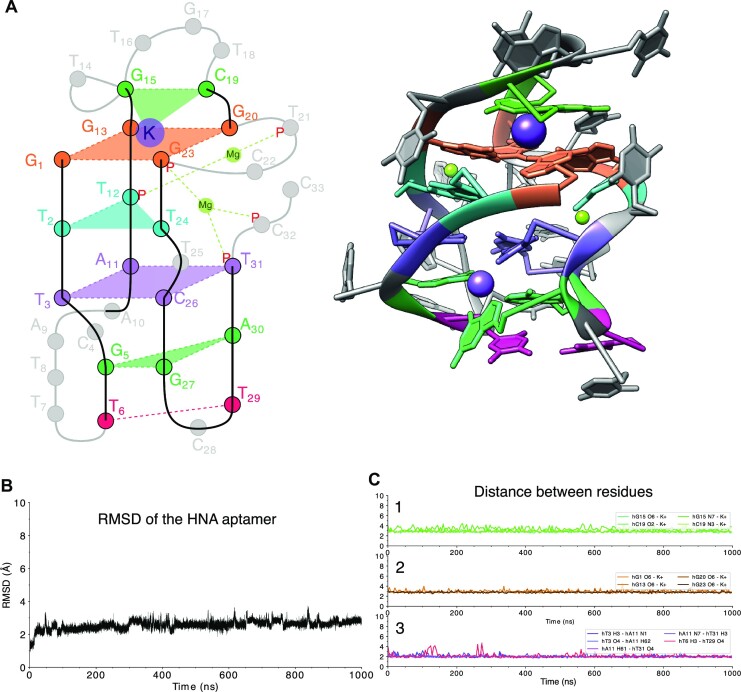
HNA aptamer model. (**A**) Proposed model of the core13 HNA aptamer after 1 μs MD in explicit solvent, showing K^+^ and Na^+^ ions (upper and lower purple spheres resp.), Mg^2+^ ions (lime sphere), G4 quadruplex (orange) and hG15-hC19 (green) interacting with K^+^), hT2-hT12-hT24 and hG5-hG27-hA30 base- triplets (cyan and see green resp.), hydrogen bonded hT6-hT29 (magenta) and planar quartet with TAT + C (lila). (**B**) RMSD (Å) during the simulation. (**C**) distance (Å) 1) between the bound K^+^ and G15-C19, 2) The K^+^ ion and the G-quadruplex and 3) the TAT + C quartet plus the hT6-hT29 interaction in the aptamer during the simulation (left) and showing residues in the constant regions (green) and consensus sequence of the variable region (yellow). (B) RMSD (Å) during the simulation (left panel) and distance (Å) between the bound K^+^ and its coordinating atoms in the aptamer during the simulation (right panel).

During the unrestrained MD simulation, the overall structure of the folded oligonucleotide remained intact. The planarity of the G-quartet stacking on proposed hTTT and hTAT triplets in a short quadruplex was well preserved (Figure 5A). Two of the nucleobases in the hTTT triplet are involved in hT-hT base pairing (45) through hydrogen bonds formed by hT2:O4-hT12:H3 and hT2:H3-hT12:O2. Base pairing of hT24 with hT12 is quickly lost in the MD, but nucleobases of these residues remain in the same plane, stacked between the G quartet and the TAT triplet in the structure. The hA11 in the proposed hTAT triplet is involved in a stable Watson-Crick base pair with hT3, and Hoogsteen interaction with hT31. Indeed, mutation of hA11 abolishes HEL binding (Supplementary Table S4). The amino group of hC26 interacts with O4 of hT31 and hT3 to form a stable hTAT + C quartet, which appears to be important for HEL binding as mutation of hT3 abolishes binding (Supplementary Table S4). A K^+^ ion remains in the central tunnel of the quadruplex, between the G-quartet and nucleobases of hG15 and hC19. The monovalent cation is coordinated by O6 of all nucleobases in the G-quartet, O6 and N7 of hG15, O2 and N3 of hC19 that closely hold the ion in place throughout the full length of the simulation (Figure 5B).

A partially hydrated Mg^2+^ ion bridges phosphate oxygens of hT12 and hT21, thereby stabilizing the short loop between hG20 and hG23. An second Mg^2+^ ion bridges this short loop with the 4′-end of the aptamer. Only the six membered sugar in hT21 (^3′^S_5′_) tends to adopt higher energy skew-like conformations at the equator of the conformational globe (ϕ = ±40° for hT21) instead of traditional chair conformations ^4′^*C*_1′_ and ^1′^*C*_4′_ residing at the North and South poles respectively (Supplementary figure S3) (47). The core of the aptamer is flanked a hG5-hG27-hA30 triplet stacking on the hTAT + C quartet. It is stabilized by hG5 and hG27′s carbonyls that coordinate a Na^+^ ion together with hC26 carbonyl of the quartet above. The nucleobase of hA30 sandwiched between hC26 and the hT6-hT29 base pair that has a stable hT:H3-hT29:O4 hydrogen bond and is formed during the MD. This stable base pairing of hT29 with hT6 (Figure 5A) appears to be important for HEL binding as mutation of hT29 abolishes binding (Supplementary Table S4). For reference, the hG27A mutation was simulated. During the MD of this mutant, the core of the structure remained stable but the Na^+^ ion was released from its pocket causing some rearrangements in the hT6-hA10 loop the 4′-tail starting with residue 27 and resulting in the loss of hT6-hT29 base pairing (Supplementary Figure S4).

## DISCUSSION

Xeno-nucleic acids (XNAs) differ from naturally occurring nucleic acids in the use of altered chemical building blocks (congeners) not observed in nature. In the case of HNA, the canonical five membered (deoxy)ribofuranose ring of DNA and RNA is replaced by a six-membered 1′,5′-anhydrohexitol ring (Figure 1A). This altered chemical configuration affords divergent physicochemical properties such as resistance to nuclease degradation and acid induced depurination (3,20) as well as altered conformational and structural preferences (37,45,47,48). While increasing success in polymerase (49) and reverse transcriptase engineering (50) has enabled a wide range of XNA aptamers as well as XNAzymes to be discovered (1,2), information on XNA structure is limited to short duplexes (19) and information on secondary and tertiary structure preferences is scarce. Apart from low resolution analysis of the secondary structure of a FANA XNAzyme revealed by chemical probing using dimethyl sulphate (DMS) (4), as well as a low-resolution structural model of a FANA octahedron obtained by electron microscopy (5), no other structural information on folded 3D XNA structures are available.

Here, we have examined the HNA aptamer (LYS-S8-19), which binds to its protein antigen, hen egg lysozyme (HEL), with nanomolar affinity. Truncation analysis revealed that shorter variants (such as LYS-S8-19-core14 (core14), LYS-S8-19-core13 (core13)) could retain and even enhance binding to HEL (Figure 1B, C). Competition with monoclonal antibody reagents and mutational mapping revealed that the HNA aptamer binding epitope does not comprise the HEL active site cleft, as originally anticipated, but rather forms a shallow and well-defined interaction surface on the antigen back face, overlapping the epitopes of the naturally occurring anti-HEL nanobody L2D24 (Figures 2 and 3), with which the HNA aptamer also shares a common interaction hotspot within this binding epitope on HEL, as well as similar binding kinetics (22).

Molecular dynamics (MD) simulations utilizing optimized force fields yielded an HNA aptamer model structure (core13) that proved stable over 1 μs of MD (Figure 5). Key characteristics of the model are the presence of a short quadruplex with a G-quartet that is stabilized by a K^+^ ion in its central channel (51) and stacking interactions of a flanking hTTT triplet and loop residues. An Mg^2+^ ion bridges phosphate moieties of hT21 and hT12 (52) that flank the hG-quartet and a second Mg^2+^ ion interacting with phosphates from hT23 and the 4′ tail. Apparently, the presence of these ions increases the conformational freedom of hexitol rings in its direct neighborhood as hT12s tends to adopt a higher energy conformers that differs from the traditional chair conformation of a six membered ring in the MD simulation(46). Strikingly, only one Watson-Crick base pair appears in the HNA aptamer structure: hT3:hA11 residing in a hTAT triplet with hT31, whereas alternative hydrogen bonding occurs among many HNA nucleobases (Figure 5A). The stabilizing effect of non-canonical base pairs is well known for natural nucleic acids (53). In HNA duplexes it was previously demonstrated for an HNA 12-mer where hT-hT base pairing prevented the sequence to form the expected hairpin structure with a T_4_-loop that was observed for DNA and RNA congeners (47). In the model proposed here, a similar hydrogen bonding pattern is observed for the non-canonical hT2-hT12 pair which is stacked between the hG quartet and hTAT triplet and hT6-hT29. Nucleobases of three residues (hT12, hG13, hG20) in the consensus motif (3) that emerged after eight rounds of selection are involved in hydrogen bonding at the stable core of the aptamer while others (hT8, hT7, hT14, hT16, hT21) are freely available for target interaction as they are exposed to the solvent.

**Figure 6.**
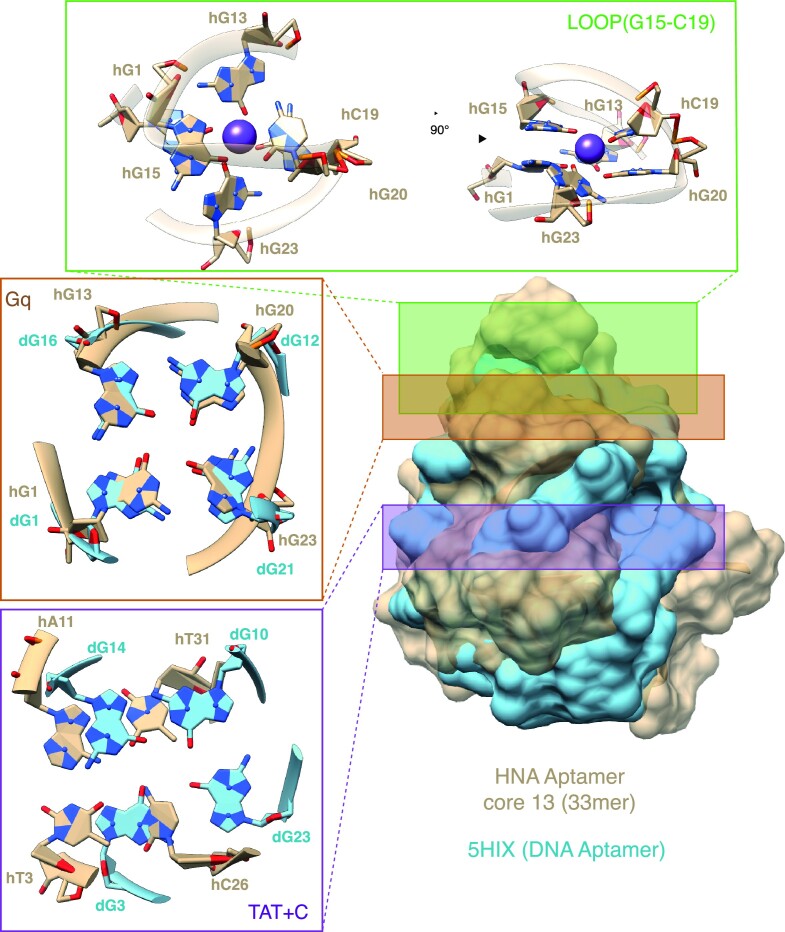
Comparisons of the HNA Aptamer model with the canonical G4 DNA quadruplex structure (PDB-entry 5HIX). (**A**) Comparison of the conceived HNA aptamer model with a known DNA aptamer [PDB ID: 5HIX]. detailed view from the top part of the HNA aptamer, showing stabilization of the G15-C19 onto the Gq (green box), the Gq_HNA_ aligned with the Gq_DNA_ (orange box) and the TAT + C_HNA_ aligning with another Gq_DNA_ (magenta box).

When overlaying the obtained model for the HNA aptamer with the canonical dG4 DNA quadruplex structure (PDB-entry 5HIX) (54) based on the G-quartet (Gq) orientation, the overall overlap in G-quartet atoms is striking (Figure 6): both nucleobases and sugar-phosphate backbone are confined within the same space with remarkable overlap which extends downstream below the G-quartet towards the hT3-hA1-hT31-hC26 quartet. This presence of an HNA G4 structure and comparable overall congruence of the HNA aptamer shape with the canonical Gq structure is further supported recognition by a well-established anti-G4 specific antibody (BG4), as well as a newly selected anti-G4 antibody (Gmab) (Figures 4 and 6). Indeed, we found HNA G4 formation (as judged by Gmab binding) to be surprisingly resilient with both K^+^ and Na^+^ ions as well as the absence of Mg^2+^ (in the presence of K^+^) supporting G4 formation (unlike in DNA) and only the presence of Li^+^ (as the only monovalent cation) abolishing it (Supplementary Figure S6).

Our model is also largely consistent with mutational analysis of core13 HEL and antiG4 Gmab binding with mutations of key residues forming the G4 structure (hG1, hG20, G23), the non-canonical hT-hA-hC-hT quadruplet (hT3, hA11) or the non-canonical hT:hT (hT29) pair abolishing binding activity (Supplementary Tables S3, S4). Surprisingly, mutation of hG13 (G13A) as part of the G4 structure abolished binding to the anti-G4 antibody, but retained some reduced HEL binding affinity, suggesting that the core13 structure in the absence of the G4 retains sufficient stability to form the anti-HEL paratope. Furthermore, we serendipitously discovered a mutation (G27A) that enhances the affinity of the HNA aptamer for HEL by ca. 7-fold, possibly by displacing a Na^+^ ion in the putative HEL paratope (Supplementary Figure S4).

The formation and unusual stability of the discussed non-canonical structures including non-canonical base-pairs may be related to the greater conformational rigidity of the HNA backbone that also greatly increases base-pairing stability. Indeed, core14 aptamer sequence synthesized in DNA showed no HEL binding suggesting that the formation of the non-canonical structures is essential to fold the HNA aptamer into its HEL-binding paratope. Indeed, loss of function by conversion of nucleic acid sequences into even very closely related chemistries is commonly observed (DNA > RNA; DNA > ANA; 2′OMe-RNA > MOE-RNA)(4,16,55), although in the case of a DNA anti-thrombin aptamer binding activity has been reported to be preserved upon conversion to PNA(56).

In conclusion, while there is a growing number of XNA aptamers and also XNAzymes, the XNA structure database currently remains limited to short duplexes(19). These include a wide range of XNAs such as deoxy(xylose) nucleic acids (XyNA), 2′-F RNA, FANA, TNA as well as pyranosyl RNA known to form non-canonical duplex structures(57–59) as well as short duplex structures for HNA oligonucleotides determined by both crystallography(37,48) and NMR (45,47). However, a description of structural configurations, folds or motifs underlying XNA function has been lacking. Our characterization and model building of the core13 HNA aptamer provides a first attempt at elucidating how a single-stranded XNA aptamer structure with fully modified backbone may fold into a three-dimensional motif to form its paratope binding both HEL and anti-G4 antibodies. While G4 structures formed from assembly of short HNA, PNA or mixed PNA-DNA oligomers have been described (60–62), this is the first G4 quadruplex structure observed in a functional motif (aptamer) formed by folding of an unbroken XNA strand. Finally, we note the striking similarities between epitopes, hotspots and biophysical binding properties between the anti-HEL HNA aptamer and naturally occurring anti-HEL antibodies underlining the conformational and structural versatility of nucleic acids and the power of *in vitro* evolution to access convergent function in chemically unrelated polymers. A better understanding of the structural diversity and functional potential of XNAs should aid further development of XNA aptamers and enzymes.

## Supplementary Material

gkad592_Supplemental_File

## Data Availability

The data underlying this article will be shared on reasonable request to the corresponding author.
