# Supplementary material for: Characterization of an HNA aptamer suggests a non-canonical G-quadruplex motif
Source: Nucleic Acids Res. 2023 Jul 13;51(15):7736–48. doi: 10.1093/nar/gkad592 (PMC10450178; doi:10.1093/nar/gkad592)
Supplement: gkad592_Supplemental_File [file gkad592_supplemental_file.pdf]

Supplementary Information

Title: Characterization of an HNA aptamer suggests a non-canonical G-quadruplex motif

Description of Supplementary Information

Supplementary Figures S1 – S6 2 – 7

Supplementary Tables S1 – S4 8 - 12

Supplementary References 13

Supplementary Figures

hCore14: 38-mer:

6’-hGhThGhChThGhThThChGhThThThAhAhAhThGhThGhThGhThChGhThChGhThThChG hChThAhThChC-4’

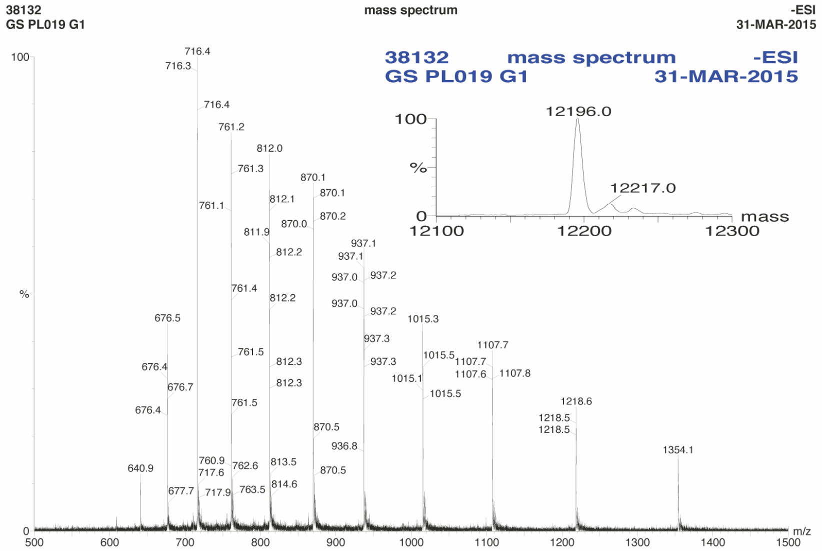

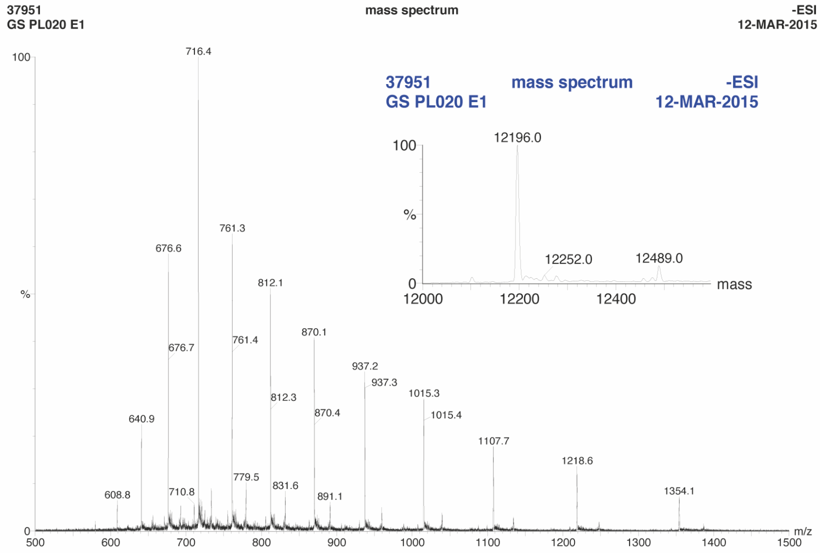

Mass average: Calcd. 12196.6
Found: 12196,0

PL 019 hG1 = 19,4 OD (purest) = 51,7 nanomol
PL 020 hG1 = 28.5 OD (pure) = 76,0 nanomol
hTotal = 47,9 OD

Supplementary Figure S1: HNA aptamer *(top) Sequence of 38-mer HNA aptamer core14 used for chemical synthesis. (middle, bottom panel) MS analysis of two batches of chemically synthesized core 14 HNA aptamer.*

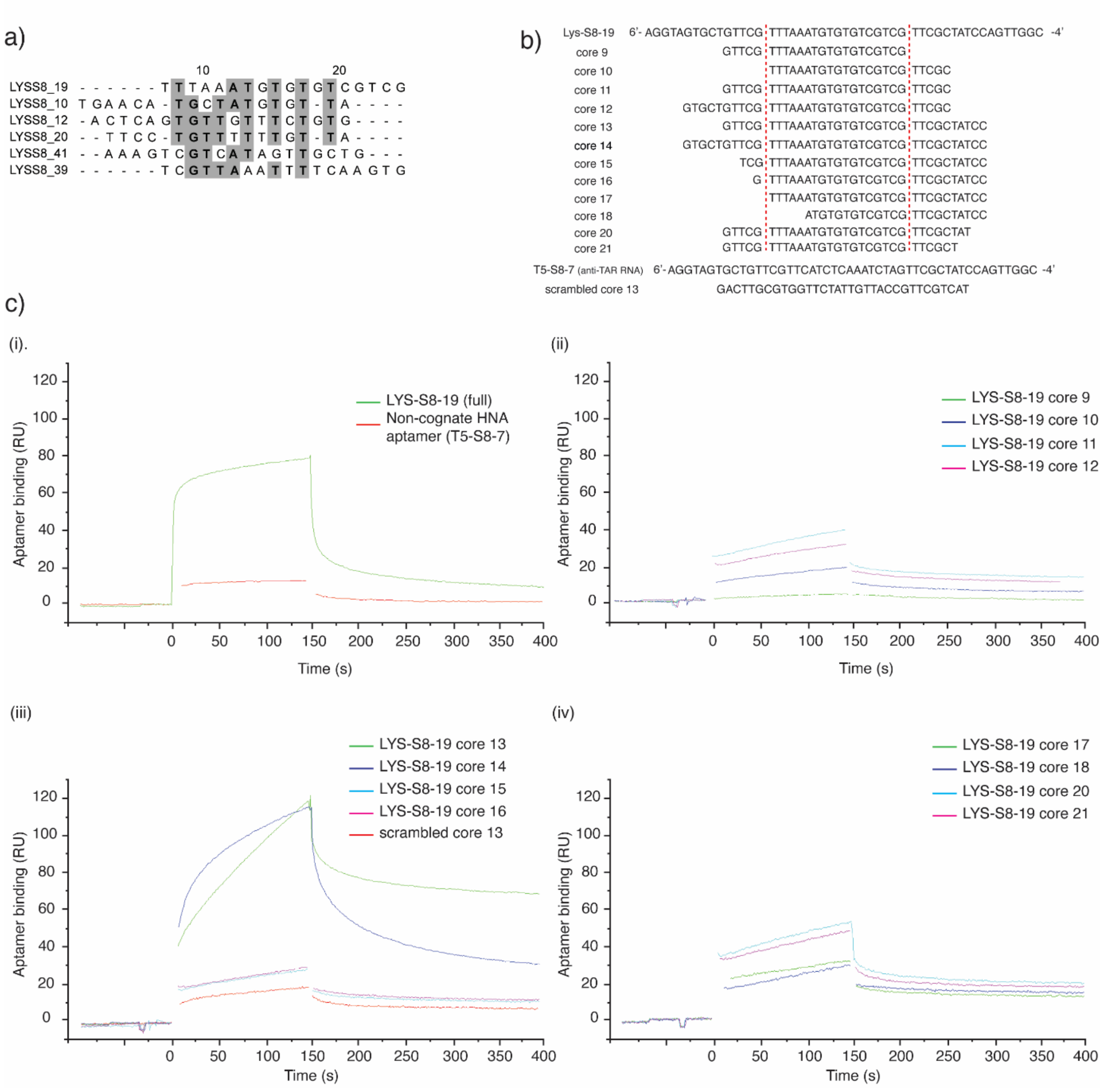

Supplementary Figure S2: *a) Alignment of anti-HEL HNA aptamer cores (tag1 and tag3 sequences omitted) showing aspects of the hGT-rich motif. b, c) HNA aptamer truncation analysis: truncated HNA sequences (b) and SPR binding curves (c) for core9-21, scrambled core13 control as well all anti-TAR aptamer T5-S8-7 controls, 50 µl 200 nM injections in buffer R, 20 µlmin*$^{-1}$ *at 20°C.*

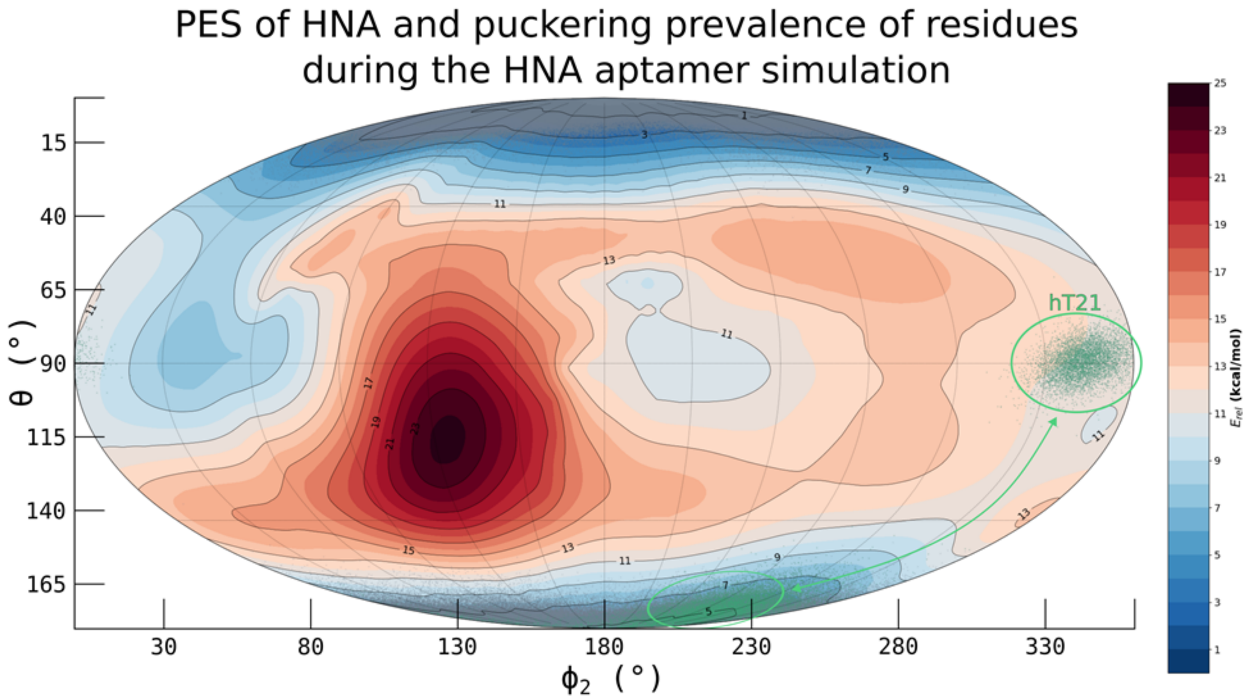

Supplementary Figure S3: *2D projection of the potential energy surface on conformational globe of HNA nucleosides, averaged over the four nucleobases (hA, hC, hG, hT). Puckering coordinates of hexitol rings in the final 50ns of the 1 μs MD simulation are represented as a scatterplot in gray dots, except for hT21 residue in dark green to denote its conformational change from a* $^{1'}C_{4'}$ *(south pole) to a* $^{3'}S_{5'}$ *(equator) .*

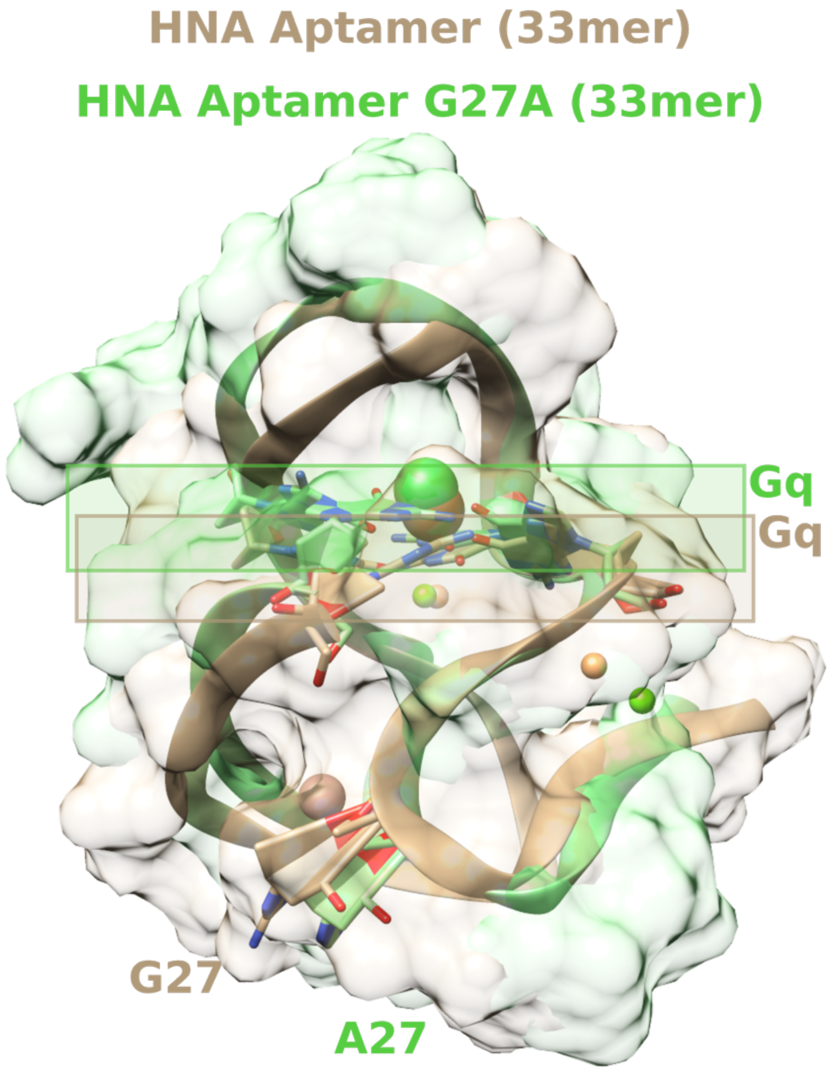

Supplementary Figure S4: *Overlay of the HNA aptamer with its G27A mutant. The original core13 aptamer is coloured beige, whereas the G27A aptamer mutant is depicted in light green. Boxes (Gq) indicate positions and superposition of the respective G-quadruplex (hG1, hG13, hG20, hG23). The original HNA aptamer contains a K^+ ion at the G-quadruplex, two Mg^{2+} ions on either side of the hT21 residue and a Na^+ ion at hG27. The mutant does not retain the Na^+ ion in the MD simulation.*

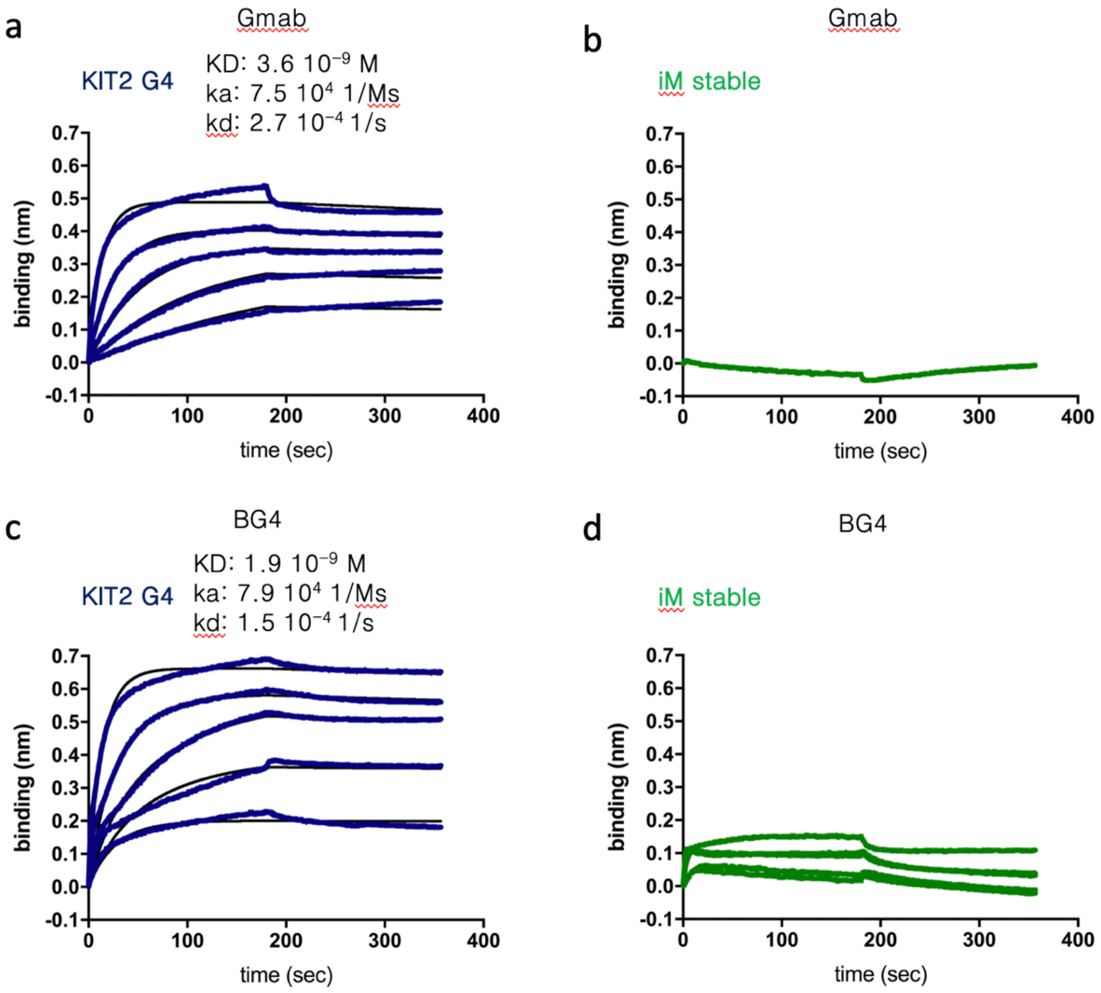

***Supplementary Figure S5: Affinity analysis of anti-G4 antibodies**. BLI affinity analysis of soluble KIT2 G4 (G4 quadruplex, blue, sequence: 5'-CGGGCGGGCGCGAGGGAGGGG) and iM stable (i-motif, green, sequence: 5'-CCCCCCCCTTTCCCCCCCC) binding to biotinylated anti-G quadruplex antibodies Gmab (a, b) and BG4 (c,d) Global fitting of kinetics revealed binding affinities of 3.6 nM and 1.9 nM for Gmab and BG4 respectively, while neither antibody had detectable binding to iM stable.*

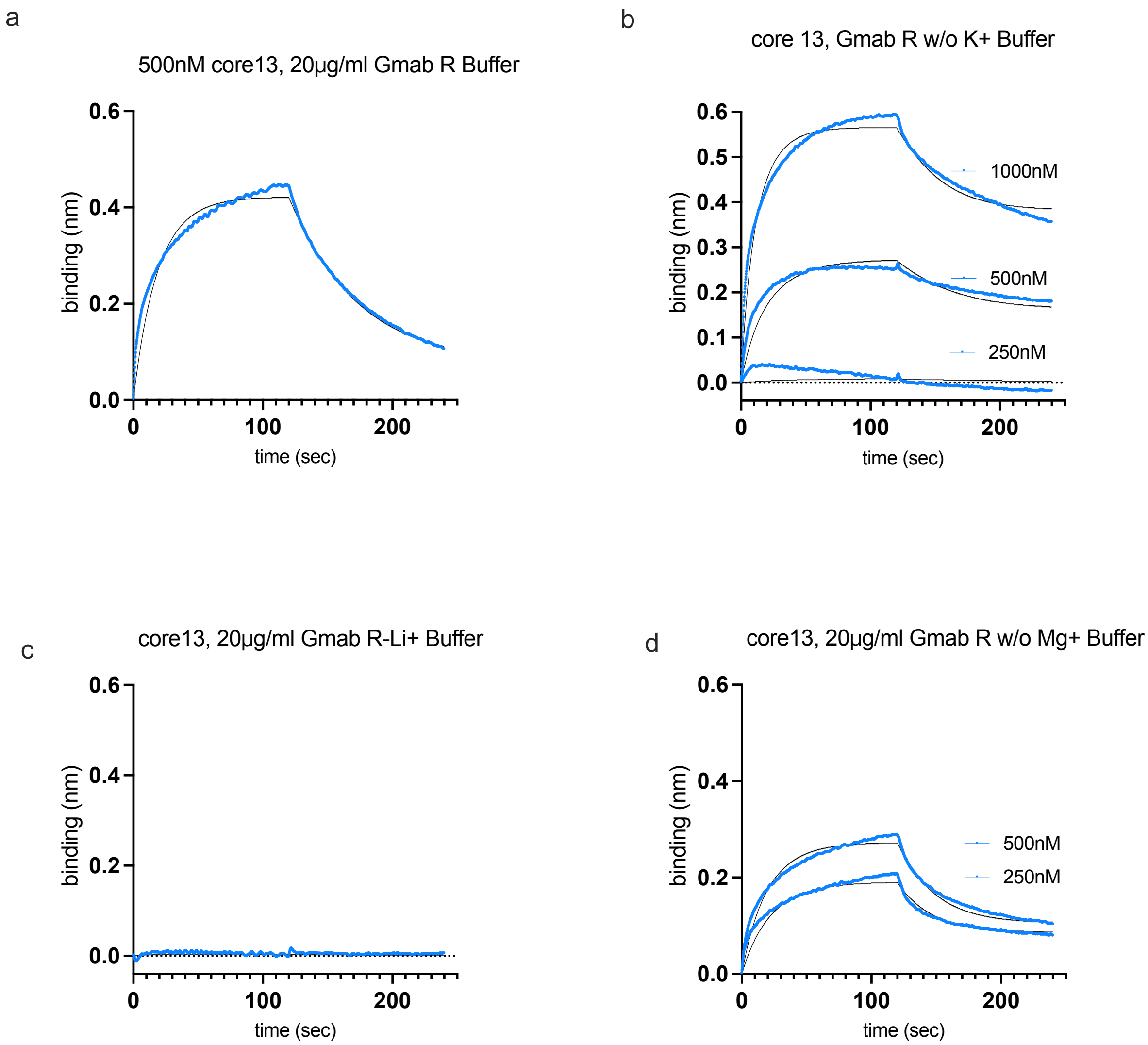

Supplementary Figure S6: HNA aptamer core13 binding to anti-G4 antibody Gmab*. a) BLI analysis of core13 binding to Gmab by BLI in buffer R: 20mM HEPES (pH 7.4), 20 mM NaOAc, 140 mM KOAc, 3 mM $Mg(OAc)_2$, 0.1% (v/v) Tween20, b) without the presence of K+ in the buffer, c) in a buffer containing Li+ and no Mg^{2+} or Na^+, d) without the presence of Mg^{2+}. Binding was measured on streptavidin biosensors loaded with 20µl/ml biotinylated Gmab. Global fitting of kinetics in b) core 13 vs Gmab without the presence of K^+ in the buffer revealed a binding of 100nM.*

Supplementary Tables

Supplementary Table S1: Oligonucleotides used for synthesis of HNA aptamer variants

Name	Sequence (5' – 3')	Notes
Cy3Fd	[CY3]CCCCTTATTAGCGTTTGCCA	Primer for HNA synthesis
LYS-S8-19temp	GCCAACTGGATAGCGAACGACGACACACATTTAAACG AACAGCACTACCTTGGCAAACGCTAATAAGGG	Template for synthesis of full-length HNA aptamer LYS-S8-19
T5-S8-7temp	GCCAACTGGATAGCGAACTAGATTTGAGATGAACGAA CAGCACTACCTTGGCAAACGCTAATAAGGG	Template for synthesis of control HNA aptamer T5-S8-7
LYS-S8-19-core9temp	CGACGACACACATTTAAACGAACTGGCAAACGCTAATA AGGG	Template for synthesis of HNA aptamer variant LYS-S8-19-core9
LYS-S8-19-core10temp	GCGAACGACGACACACATTTAAATGGCAAACGCTAATA AGGG	Template for synthesis of HNA aptamer variant LYS-S8-19-core10
LYS-S8-19-core11temp	GCGAACGACGACACACATTTAAACGAACTGGCAAACG CTAATAAGGG	Template for synthesis of HNA aptamer variant LYS-S8-19-core11
LYS-S8-19-core12temp	GCGAACGACGACACACATTTAAACGAACAGCACTGGC AAACGCTAATAAGGG	Template for synthesis of HNA aptamer variant LYS-S8-19-core12
LYS-S8-19-core13temp	GGATAGCGAACGACGACACACATTTAAACGAACTGGC AAACGCTAATAAGGG	Template for synthesis of HNA aptamer variant LYS-S8-19-core13
LYS-S8-19-core14temp	GGATAGCGAACGACGACACACATTTAAACGAACAGCA CTGGCAAACGCTAATAAGGG	Template for synthesis of HNA aptamer variant LYS-S8-19-core14
LYS-S8-19-core15temp	GGATAGCGAACGACGACACACATTTAAACGATGGCAA ACGCTAATAAGGG	Template for synthesis of HNA aptamer variant LYS-S8-19-core15
LYS-S8-19-core16temp	GGATAGCGAACGACGACACACATTTAAACTGGCAAAC GCTAATAAGGG	Template for synthesis of HNA aptamer variant LYS-S8-19-core16
LYS-S8-19-core17temp	GGATAGCGAACGACGACACACATTTAAATGGCAAACG CTAATAAGGG	Template for synthesis of HNA aptamer variant LYS-S8-19-core17
LYS-S8-19-core18temp	GGATAGCGAACGACGACACACATTGGCAAACGCTAAT AAGGG	Template for synthesis of HNA aptamer variant LYS-S8-19-core18
LYS-S8-19-core20temp	ATAGCGAACGACGACACACATTTAAACGAACTGGCAAA CGCTAATAAGGG	Template for synthesis of HNA aptamer variant LYS-S8-19-core20
LYS-S8-19-core21temp	AGCGAACGACGACACACATTTAAACGAACTGGCAAAC GCTAATAAGGG	Template for synthesis of HNA aptamer variant LYS-S8-19-core21
Core13-scramtemp	ATGACGAACGGTAACAATAGAACCACGCAAGTCTGGC AAACGCTAATAAGGG	Template for synthesis of scrambled LYS-S8-19-core13 control
Cy3Fd_1R	[CY3]CCCTTATTAGCGTTTGCCrA	Alternative primer for HNA synthesis with RNA residue (red) (hydrolysed post synthesis)

LYS-S8-19-core14[wt]temp	GGATAGCGAACGACGACACACATTTAAACGAACAGCACTGGCAAACGCTAATAAGGG[Biotin-TEG]	Alternative template for synthesis of HNA aptamer variant LYS-S8-19-core 14 (wild-type)
LYS-S8-19-core13[wt]temp	GGATAGCGAACGACGACACACATTTAAACGAACTGGCAAACGCTAATAAGGG[Biotin-TEG]	Alternative template for synthesis of HNA aptamer variant LYS-S8-19-core 13 (wild-type)
LYS-S8-19-core13[G1A]temp	GGATAGCGAACGACGACACACATTTAAACGAATTGGCAAACGCTAATAAGGG[Biotin-TEG]	Alternative template for synthesis of HNA aptamer variant LYS-S8-19-core 13 (G1A mutant)
LYS-S8-19-core13[T3C]temp	GGATAGCGAACGACGACACACATTTAAACGGACTGGCAAACGCTAATAAGGG[Biotin-TEG]	Alternative template for synthesis of HNA aptamer variant LYS-S8-19-core 13 (T3C mutant)
LYS-S8-19-core13[A11G]temp	GGATAGCGAACGACGACACACACTTAAACGAACTGGCAAACGCTAATAAGGG[Biotin-TEG]	Alternative template for synthesis of HNA aptamer variant LYS-S8-19-core 13 (A11G mutant)
LYS-S8-19-core13[G13A]temp	GGATAGCGAACGACGACACATATTTAAACGAACTGGCAAACGCTAATAAGGG[Biotin-TEG]	Alternative template for synthesis of HNA aptamer variant LYS-S8-19-core 13 (G13A mutant)
LYS-S8-19-core13[G20A]temp	GGATAGCGAACGATGACACACATTTAAACGAACTGGCAAACGCTAATAAGGG[Biotin-TEG]	Alternative template for synthesis of HNA aptamer variant LYS-S8-19-core 13 (G20A mutant)
LYS-S8-19-core13[G23A]temp	GGATAGCGAATGACGACACACATTTAAACGAACTGGCAAACGCTAATAAGGG[Biotin-TEG]	Alternative template for synthesis of HNA aptamer variant LYS-S8-19-core 13 (G23A mutant)
LYS-S8-19-core13[G27A]temp	GGATAGTGAACGACGACACACATTTAAACGAACTGGCAAACGCTAATAAGGG[Biotin-TEG]	Alternative template for synthesis of HNA aptamer variant LYS-S8-19-core 13 (G27A mutant)
LYS-S8-19-core13[G27C]temp	GGATAGGGAACGACGACACACATTTAAACGAACTGGCAAACGCTAATAAGGG[Biotin-TEG]	Alternative template for synthesis of HNA aptamer variant LYS-S8-19-core 13 (G27C mutant)
LYS-S8-19-core13[T29C]temp	GGATGGCGAACGACGACACACATTTAAACGAACTGGCAAACGCTAATAAGGG[Biotin-TEG]	Alternative template for synthesis of HNA aptamer variant LYS-S8-19-core 13 (T29C mutant)
LYS-S8-19-core14 DNA	GTGCTGTTCGTTTAAATGTGTGTCGTCGTTCGCTATCC	DNA version of LYS-S8-19-core 14 sequence

Supplementary Table S2: Restraints for Molecular Dynamics simulations.
Restraints added during each SA rounds for generating the initial model. r (Å) indicates the equilibrium distance for the harmonic restraint on interatomic distances and θ (°) the equilibrium angle for the harmonic restraint on improper dihedrals to retain planarity and reduce conformational clashes.

Round	Restraint	*r* (Å)	*θ* (°)
1	H62 (hA11) – O4 (hT3)	2.00	-
	N1 (hA11) – H3 (hT3)*	2.00	-
2	H3 (hT12) – O4 (hT2)	2.00	-
	O2 (hT12) – H3 (hT2)	2.00	-
3	H21 (hG1) – N7 (hG13)	2.00	-
	H1 (hG1) – O6 (hG13)	2.00	-
4	H21 (hG13) – N7 (hG20)	2.00	-
	H1 (hG13) – O6 (hG20)	2.00	-
	O6 (hG15) – H41 (hC19)	2.00	-
	H1 (hG15) – N3 (hC19)	2.00	-
	H21 (hG15) – 02 (hC19)	2.00	-
5	H21 (hG20) – N7 (hG23)	2.00	-
	H1 (hG20) – O6 (hG23)	2.00	-
	H21 (hG23) – N7 (hG1)	2.00	-
	H1 (hG23) – O6 (hG1)	2.00	-
6	O4 (hT12) – H3 (hT24)	2.00	-
	H3 (hT12) – O2 (hT24)	2.00	-
7	H3 (hT31) – N7 (hA11)	2.00	-
	O4 (hT31) – H61 (hA11)	2.00	-
8	H1 (hG23) – O6 (hG1) – H1(hG13) – O6 (hG20)	-	0.00
	N7 (hG1) – H21 (hG23) – N7 (hG20) – H21 (hG13)	-	0.00

Supplementary Table S3: Affinity of HNA Aptamer Variants vs biotinylated *anti-G4 antibody Gmab* Fab fragment

Top panel, HNA aptamer variants / mutants that retained anti-G4 Gmab binding activity, Bottom panel, mutants where no anti-G4 Gmab binding could be detected.

HNA aptamer variant	KD	ka	kd
Lys core13 WT	1.18×10^{-7}	8.47×10^{4}	1.00×10^{-2}
Lys core13 G27A	3.24×10^{-8}	1.36×10^{5}	4.40×10^{-3}
Lys core13 G1A	Low amplitude binding at 1000nM (poor affinity)		
Lys core13 G13A	Low amplitude binding at 1000nM (poor affinity)		
Lys core23 G20A	Low amplitude binding at 1000nM (poor affinity)		
Lys core13 G23A	Low amplitude binding at 1000nM (poor affinity)		

Supplementary Table S4: Affinity of HNA Aptamer Variants vs biotinylated HEL

Top panel, HNA aptamer variants / mutants that retained HEL binding activity (although reduced in G13A, G27C),
Bottom panel, mutants where no HEL binding could be detected.

HNA Aptamer variant	KD	ka	kd
Lys core14 WT	4.88×10^{-8}	1.81×10^{5}	8.82×10^{-3}
Lys core13 WT	6.88×10^{-8}	4.68×10^{4}	3.22×10^{-3}
Lys core13 G13A	9.79×10^{-8}	7.32×10^{4}	7.17×10^{-3}
Lys core13 G27A	9.28×10^{-9}	7.67×10^{4}	7.119×10^{-4}
Lys core13 G27C	1.16×10^{-7}	8.36×10^{4}	9.74×10^{-3}
Lys core13 G1A	Low amplitude binding at 1000nM (poor affinity)		
Lys core13 T3C	Low amplitude binding at 1000nM (poor affinity)		
Lys core13 A11G	Low amplitude binding at 1000nM (poor affinity)		
Lys core13 G20A	Low amplitude binding at 1000nM (poor affinity)		
Lys core13 G23A	Low amplitude binding at 1000nM (poor affinity)		
Lys core13 T29C	Low amplitude binding at 1000nM (poor affinity)		
Lys core14 DNA	Low amplitude binding at 1000nM (poor affinity)		

Supplementary References

1. Bhattacharyya, D., Arachchilage, G.M. and Basu, S. (2016) Metal Cations in G-Quadruplex Folding and Stability. *Front Chem*, **4**.
2. Lescrinier, E., Esnouf, R.M., Schraml, J., Busson, R. and Herdewijn, P. (2000) Solution structure of a hexitol nucleic acid duplex with four consecutive T center dot T base pairs. *Helvetica chimica acta*, **83**, 1291-1310.
3. Cash, D.D., Cohen-Zontag, O., Kim, N.K., Shefer, K., Brown, Y., Ulyanov, N.B., Tzfati, Y. and Feigon, J. (2013) Pyrimidine motif triple helix in the Kluyveromyces lactis telomerase RNA pseudoknot is essential for function in vivo. *Proc Natl Acad Sci USA*, **110**, 10970-10975.
4. da Silva, M.W. (2007) NMR methods for studying quadruplex nucleic acids. *Methods*, **43**, 264-277.
